# Effective teamwork in primary healthcare through a structured patient-sorting system - a qualitative study on staff members’ conceptions

**DOI:** 10.1186/s12875-014-0189-2

**Published:** 2014-11-28

**Authors:** Andy Maun, Miriam Engström, Anna Frantz, Elisabeth Björk Brämberg, Jörgen Thorn

**Affiliations:** Department of Public Health and Community Medicine/Primary Health Care, Institute of Medicine, The Sahlgrenska Academy, University of Gothenburg, Box 414, SE-405 30 Gothenburg, Sweden; Department of Rehabilitation Medicine Stockholm, Danderyd University Hospital, SE-182 88 Stockholm, Sweden; Närhälsan Frölunda Rehabilitation Centre, Näverlursgatan 34, SE-421 42 Västra Frölunda, Gothenburg, Sweden; Unit of Intervention and Implementation Research, Institute of Environmental Medicine, Karolinska Institutet, Box 210, SE-171 77 Stockholm, Sweden

**Keywords:** Organizational culture, Quality improvement, Teamwork, Triage, Access, Phenomenography, Primary healthcare

## Abstract

**Background:**

Primary healthcare meets increased demands from an aging population concerning quality and availability while concurrently dealing with a growing shortage of general practitioners and imperfect efficiency in healthcare processes. Reorganization and team development can improve quality and performance but projects in primary care frequently do not attain the targeted results. By developing and introducing a structured patient-sorting system a primary healthcare centre in Western Sweden increased its access rate significantly and employed its medical professionals more efficiently. The aim of this study was to explore staff members’ conceptions of the structured patient-sorting system in order to gain an inside perspective on this project.

**Methods:**

In this qualitative study 16 interviews were conducted over a period of two years and data was analysed using a phenomenographic approach to identify the various conceptions of the eleven participants.

**Results:**

Three categories of description were identified: The system was conceptualized as 1) a framework for the development of patient-centred processes that were clear and consistent, 2) a promotor of professional development and a shared ideal of cooperative practice and 3) a common denominator and catalyst in conflict management.

In an overall perspective the system was conceived as being an appropriate platform for promoting transformation into an effective patient-centred primary healthcare team in which organizational development was perceived as a continuous participative process demanding the commitment of all team members.

**Conclusions:**

This study demonstrates that the introduction of a structured patient-sorting system makes it possible for several important change processes to take place concurrently: improvement of healthcare processes, empowerment of professionals and team development. It therefore indicates the importance of an appropriate, contextualized framework to support multiple concomitant quality improvement processes. Knowledge from this study can be used to assist and improve future implementations in primary healthcare centres.

## Background

Primary healthcare in Sweden, as in many other developed countries, meets demands for quality and availability from aging populations while concurrently dealing with a growing shortage of general practitioners and imperfect efficiency in healthcare processes [1].

Prior studies show that there is growing international interest in managing organizational culture as a lever for healthcare improvement and in developing a culture emphasizing teamwork [2]. Although some light has been cast on conceptual problems with the term “culture” and there are some controversial findings, a growing line of research indicates a positive relationship between a healthcare organization’s culture and various performance measures placing emphasis on teamwork as the key cultural characteristic [2-4]. Teamwork can be understood as a dynamic process of healthcare professionals with complementary backgrounds and skills sharing common health goals and exercising concerted efforts in patient care through interdependent collaboration, open communication and shared decision-making [5]. Although team members of different professions usually collaborate sharing the same organizational routines their constructions of other professions’ roles, values and motivations can be dissonant with those professions’ constructions of themselves [6]. There is some evidence that practice-based inter-professional collaboration interventions can improve healthcare processes and outcomes [7].

Other studies have shown that the developmental stages of teams were positively correlated to improved medical outcomes and that higher team climate scores were associated with superior clinical care in diabetes mellitus, more positive patient evaluations and self-reported innovation and effectiveness [8,9].

However the major challenges are firstly that quality improvement projects in primary care frequently do not attain the targeted results but remain in their initial stages, and secondly that knowledge from evidence-informed improvement and healthcare service research remains invisible to the people who most need to use it [10,11]. Primary care practice transformation remains a demanding process requiring continual reflection, careful tailoring of interventions and on-going attention to the quality of interactions among agents in the practice [12].

At the studied Swedish primary care centre an organizational change had been conducted that aimed to combine prompt access with rational resource allocation and efficient collaboration: the introduction of a structured patient-sorting system. It originated from the idea to develop a new organizational model that combines the aims the of the Advanced Access model which has shown to reduce delays through reduction of unplanned and irrational scheduling and the Manchester Triage model which has proven reduction of waiting times and quality improvements in emergency departments [13,14]. The specific alterations of the daily processes and the quantitative results of this project have been published in an earlier article and included a 13% increase of the access rate mainly through the elimination of bottlenecks and the more efficient use of physiotherapists, psychologists and occupational therapists [15]. Both personnel and patients indicated an improvement in the possibility to book patient appointments.

Since two fundamental challenges for organizations are learning how to create effective, high-performance teams and learning how to implement and sustain quality improvements successfully, these results make it important to gain an inside perspective of the changes in the organizational culture of this specific primary care centre [16]. The aim of this study was to explore staff members’ conceptions of the structured patient-sorting system.

## Methods

### Design

In this qualitative study 16 interviews were conducted and data was analysed using a phenomenographic approach to identify the various conceptions of the eleven participants. Quality standards for qualitative studies were assured through the application of the COREQ 32-item checklist, and the main items are described below [17].

### Research team

The research team included three junior researchers (AM, AF, ME) and two senior researchers (EBB, JT). All members of the research team had a professional healthcare background covering different professions (2 physiotherapists, 1 nurse, 2 general practitioners of which one also had extensive experience as a healthcare manager) ensuring the understanding of the diverse conceptions.

### Setting

**Figure 1:**
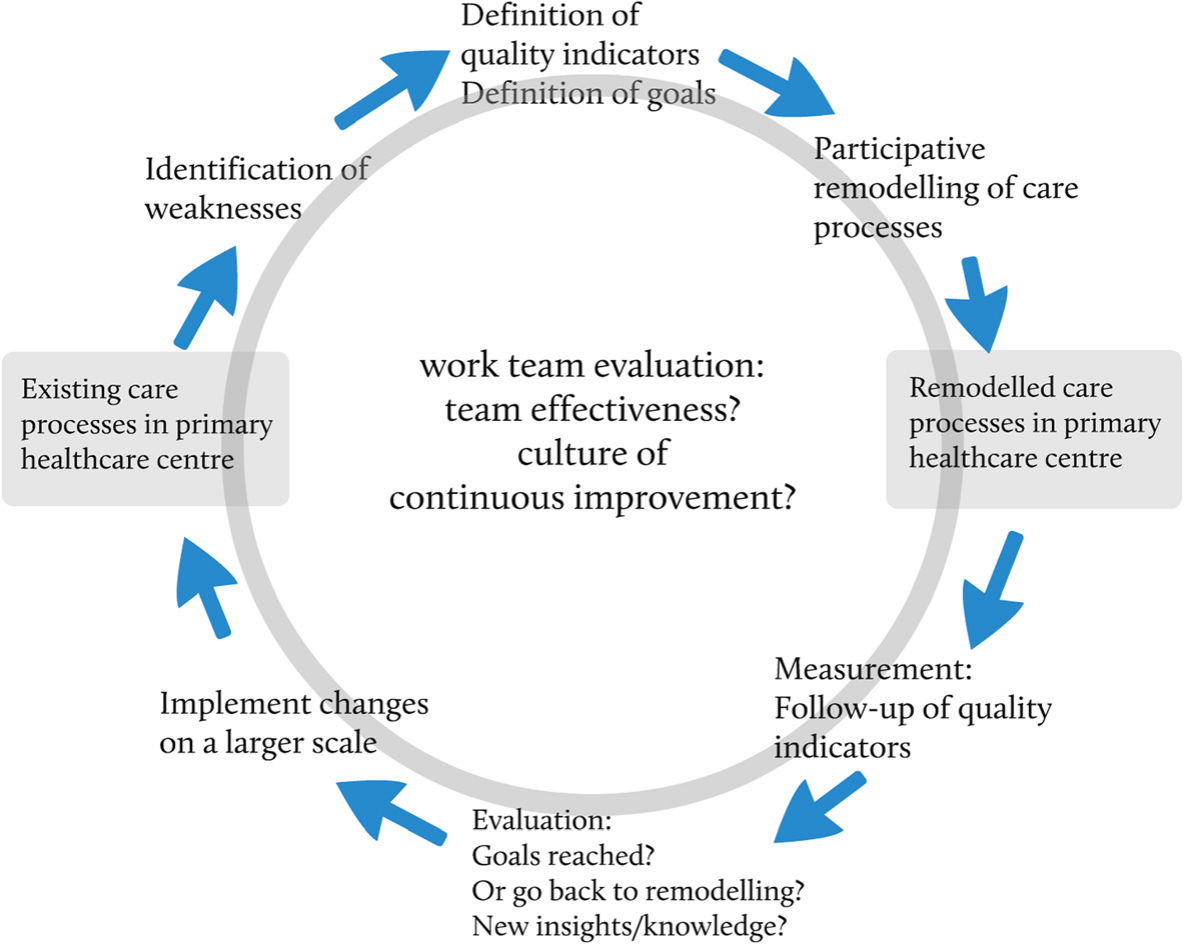
**Iterative development cycle.** The structured patient-sorting system was developed through iterative development cycles inspired by the Plan-Do-Study-Act model.

**Figure 2:**
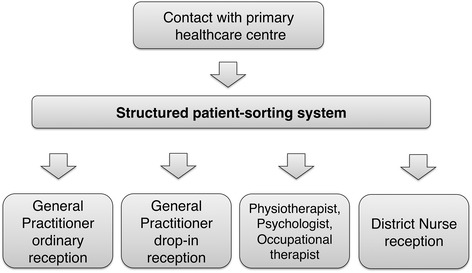
**The structured patient-sorting system.** Nurses triage all patients to the appropriate primary care professionals according to the symptoms described.

The primary care centre in Western Sweden where the structured patient-sorting system was developed and introduced in 2008 was purposely chosen for this study in order to gain a deeper understanding of the conceptions of the actively involved participants. The system was developed through iterative development cycles inspired by the Plan-Do-Study-Act model (Figure 1). All staff members participated regularly in interdisciplinary work-groups where the possible causes of the low access rate were assessed. Sorting algorithms and alterations of daily processes were developed, tested in small scale and evaluated before they where implemented for the whole primary care centre. As a result of this process nurses triaged all patients to the appropriate primary care professionals according to the symptoms described (Figure 2) in contrast to the earlier routine where most patients were initially sent to a General Practitioner. A special sorting manual was developed for this purpose in order to standardize the procedure and physiotherapists, psychologists and occupational therapists started to treat patients with certain conditions triaged directly to them by the nurses without a referral from a general practitioner [15]. Follow-up studies showed that the structured patient-sorting system seemed to satisfy the patient’s wish and need for quick access to a psychologist and that long-term healthcare consumption decreased for patients initially seeing physiotherapists [18,19]. The structured patient-sorting system has been adopted by a number of primary care centres in Sweden.

### Participants

In order to capture the various conceptions among the staff members (n = 50) the following design was chosen: all participants were strategically selected; the selection was intended to represent the full range of medical professions involved; and the inclusion criteria were active involvement in the development of and/or intensive working experience with the structured patient-sorting system. All potential participants received a written research plan outlining the purpose of the study and were informed that their participation was voluntary and the results would be handled confidentially. During recruitment only one person refused participation for reasons not related to the study. Selection resulted in a total of 11 participants (Table 1).

**Table 1:** **Participants and interviews**

**Gender**	**Profession**	**Interview 2010 duration**	**Interview 2011 duration**
f	nurse	45 min	41 min
f	nurse	45 min	45 min
f	physiotherapist	45 min	48 min
f	physiotherapist	45 min	47 min
f	manager	45 min	37 min
m	physician	45 min	---
f	nurse	---	46 min
f	nurse	---	46 min
f	district nurse	---	46 min
m	physician	---	53 min
f	psychologist	---	46 min

The sequence of participants is randomized and therefore not matching the numeration of quotes in order to guarantee confidentiality.

### Data collection

An interview guide (see ‘List of Interview Questions’ section) covering questions on different aspects of the new system was developed, tested and approved in the first interview. The participants were asked on the one hand about their conceptions of the practical structure in daily work such as new working tasks and collaboration routines and on the other hand about their conceptions of the more subtle structure such as professional roles, working climate and attitudes. In addition participants were given the possibility to enrich their descriptions by adding information on their perception of the change process itself.

**List of Interview Questions**

- What is your profession? How long have you been working here? Do you have other earlier professional experiences?
- How is working with the structured patient-sorting system? How was working here before?
- In general, what do you think about the structured patient-sorting system? Can you give examples when it worked well? What was the reason for that? Did you experience that it made work more difficult? How did you handle that?
- You started working in a new and different way, which changes were the results?
- Tasks - How did your work tasks change?
- Collaboration - How did collaboration change?
- Professional role - Has it influenced your professional role?
- How did patients respond to the new system?
- What was your most important experience with the new system?

Sixteen semi-structured interviews with an average interview length of 45 minutes were conducted and digitally recorded by AF and ME who had no prior relations to participants and no affiliation to the healthcare centre. In order to reduce the influence of current events and to ensure that conceptions were not only spur-of-the-moment ideas, six participants were interviewed one year after the full introduction of the new system and five of these again the following year. Furthermore the breadth of the various conceptions was ensured through an additional five participants who were interviewed solely in the second year (Table 1). The same interview guide was used in all interviews to prevent suggestive questioning in the follow-up interviews. The interviews were carried out at face-to-face meetings at the healthcare centre in a setting in which the participants would not be disturbed. No field notes were taken.

### Data analysis

A phenomenographic approach was used to study the various conceptions of staff members working with the structured patient-sorting system [20]. This approach has its roots in educational research and aims to characterize, understand and describe the different ways in which individuals understand the context in which they find themselves [21]. It has been used in healthcare research and literature indicates its underestimated potential for qualitative healthcare research [22].

The recorded interviews were transcribed verbatim and the quality of the transcripts was checked by the interviewers with regard to consistency with the audio recordings. The transcripts were read through several times by four members of the research team to gain an overview of the content and to obtain a sense of the whole. The transcripts were imported to the software MAXQDA 10 and then separately coded by three of the researchers (AM, AF, ME). The researchers selected and labelled utterances they found to be of interest for the question being investigated. At that stage they tried to maintain as open minded as possible to minimize any predetermined views. The three researchers individually sorted their labelled quotes that were related to each other into piles and eventually made the criterion attributes for each group explicit. During this process groups of labelled quotes were arranged, rearranged and narrowed into draft categories. At that point each of three researchers had created between 15–25 labels for on average approximately 200 quotes from the 725 minutes of interview material and had grouped the labelled quotes into 5–7 draft categories. At several meetings summing up 30 hours the three researchers discussed their individual findings and ensured validity, reliability and consistency by comparison with each other’s draft categories and labels with representative quotes and by repeated revision of transcripts. After that AM synthesized the data by writing up the findings and regularly presenting refined draft categories for the whole multidisciplinary research team for further discussion. In four two-hour meetings, that also included revisions of the transcripts when necessary, the whole team strived after a high quality of the phenomenographic outcome space by considering that each category in the outcome space revealed something distinctive about the way of understanding the phenomenon. They also ensured that the categories were logically related and that the critical variation in experience observed in the data was represented by a set of as few categories as possible [20]. Finally a negotiated consensus was reached in the research team and the phenomenographic outcome space was characterized by three categories of description supplied with representative citations. Furthermore an overall perspective of the phenomenon emerged during the final discussions. A data analysis example is provided in Table 2.

**Table 2:** **Data analysis example**

**Quotation**	**Label**	**Category of description**
*We have got much better when it comes to an understanding of each other, it’s very important to be able to cooperate in the best possible way. So you have to know what the different professions actually do in their everyday work.*	Mutual understanding of professional competences as an important component of teamwork	The system was visualized as being a promotor of professional development and a shared ideal of cooperative practice.

Data analysis example using the phenomenographic approach.

### Ethical considerations

According to Swedish law governing ethical review of research involving humans, this study did not require ethical approval [23]. Before the interviews, the participants were informed that their participation was voluntary and that they had the right to withdraw at any time without giving a reason.

## Results

### The structured patient-sorting system was perceived as a framework for the development of improved, clear and consistent patient-centred processes

The participants perceived the structured patient-sorting system as a framework where collaboration in the patient-related processes can be discussed, negotiated and determined. The participants discovered and described a demand to decrease inequalities in the booking process and to make a better use of competences and resources. They expressed the idea that the previous system was inconsistent and ineffective:

*There was an incredible breadth of expertise, … there were physiotherapists, occupational therapists, psychologists, I mean there were a lot of resources - but not used in the best possible way. (3)*

They described comprehensive achievements in patient-centred processes leading to improved efficiency and service after the implementation of the new system.

*If you look from a patient perspective this has of course led to better care. The patient will get faster to the proper caregiver… The greatest benefit is that patients get help faster. Actually the right help. (1)*

Efficiency was mainly improved through the increased use of under-employed groups in terms of competences. Physiotherapists, psychologists and occupational therapists had not previously made appointments for patients without referrals from general practitioners. When they started to make appointments for patients without referrals they stood for the sole form of treatment given in the majority of cases.

Staff members had to negotiate in a participative process on operative routines in the sorting process, which led finally to a manual with guidelines. Participants perceived that these guidelines improved patient safety through the reduction of unmotivated variation.

Some participants pointed out that the inclusion of all team members had been crucial to achieve improvements:

*The innovation meetings were good … everybody understands what is going on and everyone plays a part in it … that’s probably the most important aspect. And that all of us are equally important, otherwise it will not work. (12)*

But they also expressed the view that it had been difficult to convince members of the advantages of the new system*.* The structured patient-sorting system was not seen as a static set of routines but more as an appropriate framework for its users and their continuous development of the healthcare centre’s processes:

*We are different professionals, and therefore it’s very important that you have to update each other constantly otherwise it may drift away. … So it is in a state of constant development. (8)*

### The structured patient-sorting system was visualized as being a promotor of professional development and a shared ideal of cooperative practice

Nurses expressed the view that the new system to assess and sort the patient to the appropriate professional required more competences than the old system. They were willing to acquire this knowledge, got more confident and perceived this professional development as a positive challenge. Physiotherapists, psychologists and occupational therapists described similar experiences in relation to their expanded responsibilities when they started treating patients without referral from a general practitioner.

*If I am unsure of something, I can just go and knock on somebody’s door, which I might not have done otherwise. (5)*

*I think it has been only a positive change… you become a little bit more confident… You get acknowledgment for what you are doing, and that it works - and this is good. (7)*

These shifts of responsibility initiated a positively perceived intensification of inter-professional communication, collaboration and mutual feedback on patient cases. During these discussions team members became aware not only of differences in their approaches and views but also of the existing competences of other team members:

*We have got much better when it comes to an understanding of each other, it’s very important to be able to cooperate in the best possible way. So you have to know what the different professions actually do in their everyday work. (2)*

The participants described these inter-professional discussions as leading to something more than knowledge about each other; they mentally visualized a shared ideal of cooperative practice and appreciation of the resulting collegial relationships. The threshold for asking each other for support was lowered. The creation of value for the patient is perceived as being a task for the whole team:

*You experience more fellowship because everyone works towards the same goal: we have our patient in focus… This makes you see the big picture and feel that we’ll fix everything together. (12)*

Even other professionals outside the healthcare centre noticed and commented upon positive changes, resulting in further positive impact on the self-image of the team.

### The structured patient-sorting system was envisaged as being a common denominator and catalyst in conflict management

One of the most obvious differences before and after the implementation of the new system is the fact that some staff members had previously operated almost independently from the rest of the healthcare centre and now suddenly all staff members were expected to become part of the larger team which was a cause for conflicts.

*It was like war – before they (the district nurses) did not have had this at all (referring to the open reception). They said that all of them would resign, and it was terrible! And then once they had tried it and had worked with it, they finally got the feeling that they also could decide how it should be and thought it was good. Now they think it’s great. (9)*

Participants described the existence of latent conflicts between subgroups and individuals especially if they had been working rather in isolation from each other. During the implementation of the structured patient-sorting system these latent conflicts became of necessity visible and led to open confrontations.

*These conflicts existed, of course, also before - that one felt that a patient has been booked incorrectly. But now there was a platform to discuss it. […] So earlier it was like talking that did not lead to a change, but now it is like: “Ok, next time when such a patient comes we are going to handle it in that way…” (4)*

The management of the healthcare centre created forums for conflict management where the whole team met on a regular basis to discuss how the patient-sorting system was currently working and whether or not the need existed to adapt or develop it further. At these meetings confronting views could be expressed aloud and the team tried to solve the conflicts through direct communication.

*When I see it in the booking calendar I can now tell a nurse who booked a patient to a doctor: “Why didn’t you send this patient to me? This is definitely a patient who should be sorted to a physiotherapist”. “But this patient had a swollen knee”. “Ok, but we have agreed that patients with knee-pain should initally come to me regardless if it’s swollen or not” (1)*

Even if these conflict management meetings were perceived as demanding and time-consuming, team members expressed a positive attitude towards them and endorsed the opportunity to solve the conflicts through communication:

*I know there are many who find it tough to go to meetings and talk about everything. The downside is that it takes a lot of time but it is always like this - there’s no system that’s totally perfect. … You need not be concerned that conflict is always negative. There will be always conflicts when you have people discussing issues with each other. You should try to see the positive side of the conflict instead. … And it’s a very democratic system that we have here where all issues must be raised and discussed. Yes, I think it’s good actually. (10)*

For different reasons like impending retirement or reluctance to collaborate with other team members some staff members disliked the changes. Thus participants perceived the structured patient-sorting system as a catalyst in a selection process: the majority of staff members experienced an active ownership of the structured patient-sorting system and felt encouraged while a few staff members disliked the changes and eventually decided to leave the team. Interestingly the participants did not express negative feelings about the loss of these staff member but accept it as a part of the process.

*I do not think there was much resistance, but it was harder to introduce an open reception for one group who had previously organized their reception entirely by themselves. Those who did not agree with the change quit, and so it was like a small self-regulation, which was pretty good - which was needed here. (9)*

*That employee quit. I kept on saying all (team members) were like new after that change, as a part of the process. (2)*

The participants formulated the idea that the management’s leadership and communication played an important role in conflict management. They visualized the leadership model as a balance between openness and sensitivity on the one hand and responsiveness and consistency on the other hand. It was vital for the management to be present at all times in order to mediate and execute minor adaptations and to make sure that all team members complied with the negotiated routines.

#### Relations between categories of description and overall perspective

The three categories of description reflected different ways of understanding of the phenomenon: 1) A rather technical understanding, 2) an understanding of identity on both an individual but also on a group level and 3) an understanding of interdependency and the complex dynamics of the system. These three ways of understanding were equally important and complementary. During the final discussions with the whole research team an overall perspective on the interview material emerged:

The structured patient-sorting system was conceived as being an appropriate platform for promoting transformation into an effective patient-centred primary healthcare team in which organizational development was perceived as a continuous participative process demanding the commitment of all team members.

The analysis accounted for above reveals clearly that several change processes took place concurrently and that it was rather a transformation of the whole healthcare centre than a sum of incremental changes. The development and introduction of the structured patient-sorting system required the active inclusion of all team members and did not leave members emotionally unaffected. Through continuous adaptations and a management with permanent presence and explicit responsiveness the team could finally develop a collective responsibility aiming to find the best possible solutions for their patients by utilizing all resources in the team effectively.

## Discussion

This study shows that staff members formed a concept of the structured patient-sorting system as an appropriate platform for the organizational transformation process into becoming an effective team. Several change processes were handled concurrently: the improvement of healthcare processes, the empowerment of professionals and team development. Overall the transformation was perceived as a continuous participative process demanding the commitment of all team members.

The phenomenographic approach fits this qualitative study well as it allowed us to capture how participants from different medical professions experienced the introduction of the structured patient-sorting system in different ways, thus forming different conceptions of it. By repeating the interviews in the study after one year, the results were stabilized and the risks reduced that current events might have influenced the impressions of the participants. The gender-balanced, multidisciplinary research team that held frequent discussion sessions during the analysis process contributed to ensuring that all the varied conceptions of the participants could be identified. The fact that the research team consisted both of members who had worked at the site investigated and members with no affiliations to the healthcare centre is yet another of the study’s strengths: it facilitated an understanding of complex internal processes and at the same time ensured a distanced viewpoint on the interview material. Interviews with all staff members instead of the eleven participants might possibly have resulted in even more conceptions but were practically impossible to perform due to resource limitations having in mind that the collected material is already extensive. Interviews with former staff members who had left the team might have uncovered even more aspects of the change process but were for practical reasons too difficult to perform as some had moved to other places. The inclusion of interviews with patients might have contributed further perspectives but that was not focus of this study. Another limitation of this study is the fact that there were other external changes during the time of the study (i.e. a regional healthcare reform) that might have influenced the participants’ ideas even if none of them mentioned it explicitly.

The findings of this study correspond well to earlier research on high-performance teams and their characteristics: collaboration, conflict resolution, participation, and cohesion were most likely to influence staff satisfaction and perceived team effectiveness [24]. The characteristics of an effective network for improvement apply well to the team studied: common purpose, cooperative structure, critical mass, collective intelligence and community building [25].

This study shows how it may be possible to meet one of the major challenges of quality improvement: to go beyond initial stages of a project and reach the targeted results [10,16].

Previous research has shown that quality improvement agents need to know how new working methods and procedures are implemented, understand the target groups and the setting, try to see target groups’ perspective and involve them in both the development and implementation of the innovation [26]. In addition it is known that a well-organized implementation process will contribute to successful implementation, overcoming many barriers and unhelpful factors. Moreover, continuous evaluation of the actual care process and monitoring of the changes are also crucial in the ensuing success of the implementation activities.

The conceptualization as an appropriate platform for the transformation to an effective team indicates the importance of a contextualized framework that supports the concomitant implementation of multiple quality improvement processes. The system is bridging more elusive issues like attitudes and professional identities to more concrete and practical ones such as booking routines or areas of responsibility. Negotiation on how tasks need to be solved leads to more clarity and the development of the cooperative relationships. There was only a low degree of disengagement over time, which indicates that content of the regular team meetings was perceived as relevant. However, it must be stated explicitly that the structured patient-sorting system in itself cannot be seen as a guarantee for a successful transformation without demanding and time-consuming work involving all team members.

Even if the scope of this study is limited to one healthcare centre its findings can be transferred to and re-used in future implementations in other healthcare centres, since prior research has shown that experiences from similar change initiatives proved to be a helpful success factor in new implementations [27]. Further studies of both successful and failed quality improvement projects are needed to gain more knowledge on the underlying success factors for change.

## Conclusions

This study demonstrates that the introduction of a structured patient-sorting system made it possible for several important change processes to take place concurrently: improvement of healthcare processes, empowerment of professionals and team development. It therefore indicates the importance of an appropriate, contextualized framework that supports multiple concomitant quality improvement processes. Knowledge from this study can be used to assist and improve future implementations in primary healthcare centres.
